# Derivation and elimination of uremic toxins from kidney-gut axis

**DOI:** 10.3389/fphys.2023.1123182

**Published:** 2023-08-15

**Authors:** Ying Xu, Wen-Di Bi, Yu-Xuan Shi, Xin-Rui Liang, Hai-Yan Wang, Xue-Li Lai, Xiao-Lu Bian, Zhi-Yong Guo

**Affiliations:** ^1^ Department of Nephrology, Changhai Hospital of Naval Medical University, Shanghai, China; ^2^ Brigade One Team, Basic Medical College, Naval Medical University, Shanghai, China

**Keywords:** uremic toxins, derivation, elimination, gut microbiome, natural products

## Abstract

Uremic toxins are chemicals, organic or inorganic, that accumulate in the body fluids of individuals with acute or chronic kidney disease and impaired renal function. More than 130 uremic solutions are included in the most comprehensive reviews to date by the European Uremic Toxins Work Group, and novel investigations are ongoing to increase this number. Although approaches to remove uremic toxins have emerged, recalcitrant toxins that injure the human body remain a difficult problem. Herein, we review the derivation and elimination of uremic toxins, outline kidney–gut axis function and relative toxin removal methods, and elucidate promising approaches to effectively remove toxins.

## 1 Introduction

Uremic toxins are described as organic or inorganic chemical substances, that accumulate in the body fluids of individuals with acute or chronic kidney disease (CKD) and renal dysfunction (Glassock et al., 2022). They are metabolites that are generated daily from food by metabolism and excreted into the urine through glomerular filtration or active transport by renal proximal epithelial cells (Vanholder et al., 2003), which may cause uremic syndrome due to the cumulative effect of these disturbances in renal elimination and subsequently, toxicity (Meyer and Hostetter, 2007; Almeras and Argilés, 2009). Up to 75 individual clinical symptoms, including memory and cognitive dysfunction, asthenia, headache, confusion, anorexia, gastroparesis, hematologic anemia, hemostatic disorders, hypertension, atherosclerosis, coronary artery disease, pruritus and skin dryness, calciphylaxis, growth impairment, impotence, infertility, sterility, osteomalacia, β2-microglobulin amyloidosis, increased susceptibility to infection, metabolic acidosis, hyperphosphatemia, hyperkaliemia, and many other diseases (Almeras and Argilés, 2009), may lead to damage to every organ, a reduced quality of life and an increase in morbidity and mortality.

The most thorough reviews to date, prepared by the European Uremic Toxins Work Group (EUTox), list no less than 130 uremic solutes, and new studies continue to add to this number (Vanholder et al., 2003). Based on molecular weight and chemical characteristics, uremic toxins are divided into three categories. The first category consists of free, water-soluble, low-molecular-weight solutes (<500 Da), for example, creatinine and urea, which are readily and efficiently eliminated by conventional dialysis. The second, middle molecules (≥500 Da), include peptides and proteins, with representative examples being β2-microglobulin and α1-macroglobulin, which can only be eliminated by dialysis treatment performed using dedicated dialyzer and are characterized by larger pores on the membrane surface due to their molecular weight. Last but not least, protein-bound solutes are often referred to as protein-bound uremic toxins (PBUTs), which have a high binding affinity for albumin or other carrier proteins, which severely disturbs the clearance rate of conventional hemodialysis (HD) treatments, even with high-flux methods (Vanholder et al., 2003; Vanholder et al., 2008; Wu et al., 2011; Duranton et al., 2012; Itoh et al., 2012; Viaene et al., 2013; Chmielewski et al., 2014; Clark et al., 2019).

Given the complex and difficult quantification of uremic toxins, it is a daunting task to match an isolated solute or a set of solutes to a definite symptom. Such difficulty is imposed by a variety of solutes and compounded by the multiplicity of ill effects encountered in uremia (Meyer and Hostetter, 2007). At present, the prevailing viewpoint is that certain unexplained symptoms of uremia are largely caused by the accumulation of organic wastes due to the dysfunction of renal removal processes. Because little is known about the toxicity and mechanisms of uremia toxins in the human body when they act alone or in combination, the study of these toxins has been focally organized on the basis of their source and elimination.

## 2 Generation of uremic toxins

**FIGURE 1:**
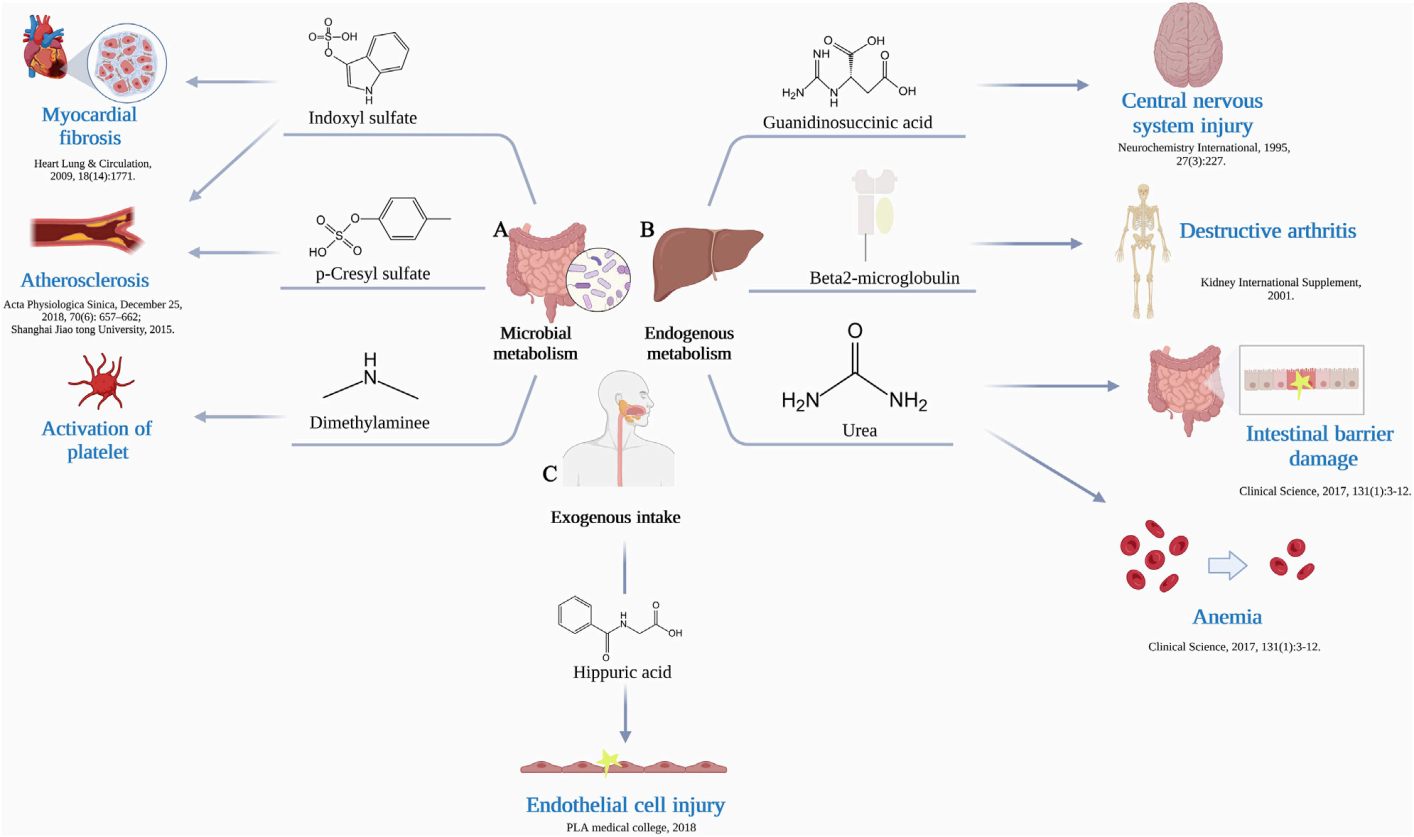
**(A)** The gut microbiome generate uremic toxins. Production of indoxyl sulfate by gut microbiome leads to myocardial fibrosis and atherosclerosis, production of p-Cresyl sulfate leads to atherosclerosis, production of dimethylamine leads to activation of platelet. **(B)** Endogenous metabolism by liver produce uremic toxins. Production of guanidinosuccinic acid leads to central nervous system injury, production of β2-Microglobulin leads to destructive arthritis, production of urea leads to intestinal barrier damage and anemia. **(C)** Exogenous intake supplies a source of substrates. The resulting production of hippuric acid causes endothelial cell injury.

Progress in medicine with the extended applications of gas chromatography, mass spectrometry, and high-performance liquid chromatography has altered the way in which is it determined if a serous solution is toxic. Nevertheless, the problem of tracing the metabolism of a solute and its renal and systemic toxicological effects remains. Thus, unraveling the source of uremic toxins could help to find new ways to remove them more efficiently. Each uremic solute may have multiple sources, although we focus on only one in this review. The pathways of uremic toxin production are shown in Figure 1 and Table 1.

**TABLE 1:** Derivation and mechanism of uremic toxins.

Uremic toxins Meyer and Hostetter (2007), Graboski and Redinbo (2020)								
Group	Indoles	Phenols	Aliphatic amines	Hippurates	AGEs	Peptides and small proteins	Guanidines	Nucleosides
Example	Indoxyl sulfate	p-Cresyl sulfate	Dimethylamine	Hippuric acid	Carboxymethyllysine	β_2_-Microglobulin	Guanidinosuccinic acid	Pseudouridine
Derivation	Microbial metabolism	Microbial metabolism	Microbial metabolism	Exogenous intake and microbial metabolism	Exogenous intake	Endogenous metabolism	Endogenous metabolism and microbial metabolism	Endogenous metabolism
Pathogenies/Mechanism	Increased expression of genes linked to tubulointerstitial fibrosis Miyazaki et al., (1997), vascular stiffness Adijiang et al., (2010), aortic calcification Adijiang et al., (2010), and cardiovascular mortality Barreto et al., (2009); oxidative stress in endothelial cells Dou et al., (2004); vascular smooth muscle cell proliferation Adelibieke et al., (2014); nephrotoxicity Niwa and Ise, (1994); Miyazaki et al., (1997)	Further development of CKD, cardiovascular disease (CVD), and mortality in hemodialysis patients Liabeuf et al., (2010); Wu et al., (2011); decreased production of endothelial adhesion molecules when cytokines are present Dou et al., (1999); endothelial permeability (Neirynck et al., 2012)	Platelet activation Gao et al., (2021); neurotoxicity, hemolysis, and lysosomal function inhibition Adijiang et al., (2010)	Nontoxic; increased anion gap acidosis; may lead to glucose intolerance and impair the functions of platelet cyclooxygenase and erythropoiesis Ramezani et al., (2016)	NF-κB/MAPK/JNK signaling; extracellular matrix (ECM) formation of crosslinks; impaired endothelial progenitor cell function; RAGE signaling Ma et al., (2023)	Inappropriate activation of various hormone or cytokine receptors; systemic inflammation; accelerated vascular disease Adijiang et al., (2010)	Uremic platelet dysfunction; central nervous system dysfunction; decreased neutrophil function Luft, (2008)	

CMPF, 3-carboxy-4-methyl-5-propyl-2-furanpropionic acid; AGE, advanced glycation end product.

### 2.1 Uremic toxins from the gut microbiome

Over the past 2 decades, research on the interactions between the host and the microbiota has grown substantially, and it is now apparent that commensal bacteria are involved in the regulation of numerous physiological processes in the host (Schroeder and Bäckhed, 2016). The gut microbiome, which performs a variety of tasks and can be thought of as a metabolically active endogenous ‘organ’, is the collective microbial genome of the gut microbiota (Ramezani and Raj, 2014a). Under physiologic conditions, the digestion of food, development of host immunity, control of gut endocrine function and neurological signaling, alterations in drug action and metabolism, removal of toxins, and production of various chemicals with properties that influence the host have all been attributed to the gut microbiome (Fan and Pedersen, 2021). Beyond these, the gut microbiome engages in a number of complementary metabolic processes that are not fully developed in the human host, including the breakdown of undigestible plant polysaccharides (Hooper et al., 2002), synthesis of certain vitamins (Hill, 1997), biotransformation of conjugated bile acids (Hylemon and Harder, 1998), and degradation of dietary oxalates (Duncan et al., 2002; Ramezani and Raj, 2014a). For instance, using the individual-specific and temporally stable microbial profiles, including bacterial SNPs and structural variations, Chen et al. develop a microbial fingerprinting method. When observing the microbial associations with metabolites, there were 27 associations related to different uremic toxins, especially hippuric acid. (Chen et al., 2021).

The gut microbiome can generate uremic toxins from various substrates, including amino acids and the choline class of compounds (Wang et al., 1002). The bacterial proteolytic fermentation process in the large intestine accounts for most of the potentially toxic end products. This is also why most gut-derived toxins are nitrogenous compounds. In the process of fermentation, several factors are responsible for the mechanism and are increased by CKD, which creates a latently different intraluminal environment, including the ratio of carbohydrates to proteins, colonic transit time, and bacterial composition in the intestines (Evenepoel et al., 2009). The gut microbial metabolic toxins that are often mentioned and studied include indoles, phenols, aliphatic amines, and polyols (Table 1) (Figure 1A). Many colon-derived solutes metalized by the microbiome are PBUTs (Mair et al., 2018). Indole and its derivatives are directly transformed by intestinal microorganisms in the gut through tryptophan metabolism (Agus et al., 2018), many of which are ligands for the aryl hydrocarbon receptor (Zelante et al., 2013). Tryptophan is first converted into indole by gut microbial tryptophanase enzymes, and then indole is transported to the liver. Then the tryptophan is hydroxylated and sulfated by human hepatic cytochrome P450s and sulfotransferase enzymes, which form the circulating and harmful uremic toxin indole sulfate (IS) (Banoglu et al., 2001), respectively. Moreover, fermentation of aromatic amino acids may generate a variety of bioactive end products, such as phenol and p-cresol (tyrosine) (Rowland et al., 2018). P-Cresyl sulfate (pCS) is one of the most thoroughly studied and harmful phenolic uremic toxins, which is produced through multiple steps involving intestinal microbial and host liver factors (Graboski and Redinbo, 2020). Gut microbial enzymes convert tyrosine to p-cresol either directly through tyrosine lyases or through a multistep process involving tyrosine transaminases and 4-hydroxylphenylacetate decarboxylases (Saito et al., 2018). A small portion of the p-cresol produced by the gut stays in the gut and is transformed into p-cresyl glucuronide by host epithelial UDP-glucuronosyltransferase, while the majority of the p-cresol is absorbed into systemic circulation (Graboski and Redinbo, 2020). Then, hepatic sulfotransferases transform the p-cresol that enters systemic circulation into the uremic toxin pCS (Gryp et al., 2017). The study by Gryp et al., 2020, suggests that although the increase of plasma levels of PBUTs produced in the intestine is mainly a result of renal dysfunction, gut bacteria remain an important potential target when considering new treatment methods to prevent the accumulation of uremic toxins. (Gryp et al., 2020).

### 2.2 Uremic toxins from endogenous metabolism

As early as 1964, Dawes proposed that endogenous metabolism is simply the sum of the metabolic processes that take place inside of a living organism when there are no substances or materials acting specifically as exogenous substrates. The liver is the essential organ that governs a substantial portion of metabolism and serves as a hub to link diverse metabolic pathways. Since urea was the first organic solute identified in the blood of patients with kidney failure, it is quantitatively the most significant solute eliminated by the kidney, with both HD and peritoneal dialysis (PD) currently being prescribed to achieve target values for urea clearance (Meyer and Hostetter, 2007). In the liver, to produce one molecule of urea, two ammonia molecules and one CO_2_ molecule are shuffled into the cycle. One ammonia molecule combines with carbon dioxide and the currently available precursor from the previous ornithine cycle to subsequently form citrulline. The other ammonia is then combined to form arginine. Arginine is known to take part in the production of creatine, thereby boosting energy and reducing exhaustion, similar to methylguanidine. Uric acid and xanthine are also endogenous molecules produced without interference by intestinal absorption (Gouroju et al., 2017). Guanine monophosphate is converted into guanosine by nucleotidase, which, together with a nucleoside and inosine, is further converted to the purine-based compounds hypoxanthine and guanine by purine nucleoside phosphorylase. Hypoxanthine is oxidized by xanthine oxidase to form xanthine, and guanine is deaminated by guanine deaminase to produce xanthine. Xanthine is then again oxidized by xanthine oxidase to form the final product, uric acid. Additionally, a few scholars have argued that uremic toxin synthesis may originate from mitochondria, which closely ties the function and generation of uremic toxins to mitochondrial metabolism, possibly through key biochemical pathways (Popkov et al., 2019). For example, creatinine (or closely related creatine) metabolism is tightly associated with mitochondria (Wyss and Kaddurah-Daouk, 2000); organic acids, including argininic acid, hippuric acid, indole-3-acetic acid, orotic acid, α-keto-δ-guanidinovaleric acid, γ-guanidinobutyric acid, uric acid, and kynurenic acids, represent another group of uremic toxins, the metabolism of which is dependent on mitochondria; and some nucleotide derivatives, uric acid, xanthine, hypoxanthine, urea, and phenylacetylglutamine, are closely associated with mitochondrial metabolism (Popkov et al., 2019) (Figure 1B).

### 2.3 Uremic toxins generated from exogenous intake

Exogenous intake, the gut microbiome and endogenous metabolism are three intersecting sources of uremic toxins that are inseparable (Figure 1C). Exogenous intake supplies a source of substrates, many of which provide substances that the human body cannot naturally synthesize. Hippurate, a kind of uremic toxin, is primarily obtained from plant-based foods, as only a small quantity is produced endogenously from the amino acid phenylalanine (Brunelli et al., 2021; De Simone et al., 2021). Diet therefore determines hippurate production, a process in which Clostridia spp. are involved (Ramezani et al., 2016). In addition, furans, advanced glycation end products (AGEs), and polyols are also produced in this way (Gouroju et al., 2017). AGEs are heterogeneous compounds formed through a nonenzymatic Maillard reaction sequence in which reducing sugars are covalently linked to protein amines, most commonly lysine and arginine residues (Stinghen et al., 2016). Fructoselysine, methylglyoxal, glyoxal, and 3-deoxyglucosone are the precursors of circulating AGEs, and the majority of these precursors are byproducts of various metabolic and oxidative processes, such as glycolysis, lipid peroxidation, and the breakdown of glycolytic intermediates (Abordo et al., 1999). The modern diet is full of dangers that greatly contribute to the body’s AGE pool, especially when cooked at high heat in dry conditions (Mallipattu et al., 2012; Snelson and Coughlan, 2019). Examples include cereal, baked goods, and powdered milk, which raise systemic AGE levels.

## 3 Metabolism and elimination of uremic toxins

**FIGURE 2:**
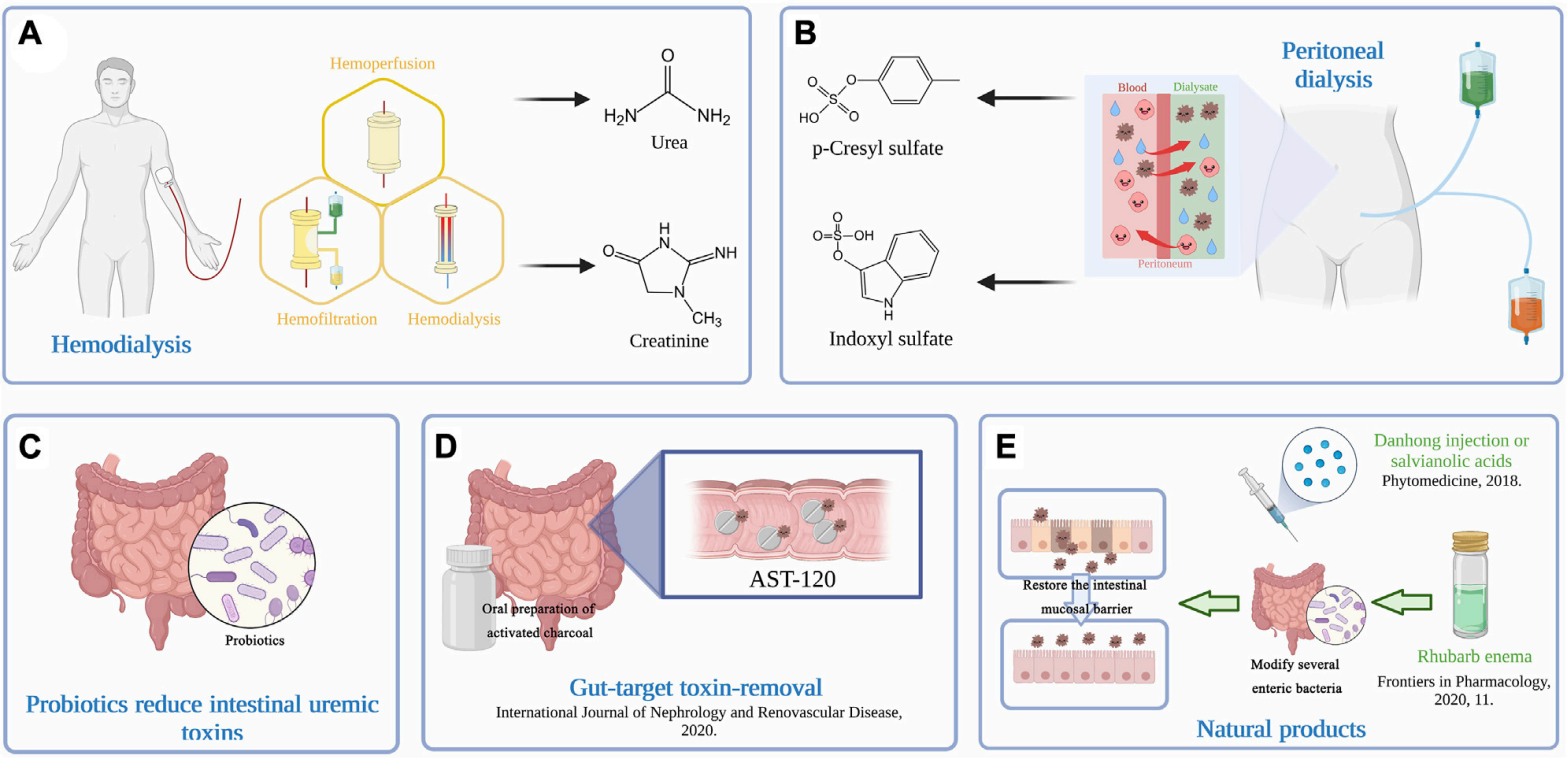
**(A)** Extracorporeal hemodialysis techniques. Therapeutic HD removes solutes primarily by adsorption, diffusion, and convection across semipermeable membranes. **(B)** Peritoneal dialysis removes excess water and toxins via the peritoneum, a natural semipermeable membrane, and provides more flexibility and independence for patients. The understanding of solute and water transport processes across the peritoneum underlies the efficiency of PD. **(C)** Probiotics act by reducing kidney injury and fibrotic-related proteins, decreasing oxidative stress and proinflammatory reactions and elevating immune responses, reversing gut dysbiosis, and restoring the abundance of commensal bacteria. **(D)** Intraluminal adsorption onto high-affinity surfaces may also serve as an effective intervention for toxin removal. An oral preparation of activated charcoal, AST-120 has demonstrated its effectiveness in toxin removal. **(E)** Natural products have been considered a promising and effective treatment for ESRD. Rhubarb enema treatment could restore the intestinal mucosal barrier by modifying several functional enteric bacteria. The use of Danhong injection or salvianolic acids inhibits CKD progression by removing uremic toxins.

Currently, as fundamental and life support therapies, traditional HD and PD are still the mainstream methods by which uremic toxins are removed from the body. HD is one of the common methods of renal replacement therapy for toxin removal. Material exchange is carried out through the principles of dispersion, ultrafiltration, adsorption and convection to remove metabolic wastes, maintain the electrolyte and acid-base balance, and remove excess water from the body. Both HD and PD are currently prescribed to achieve target values for urea clearance (Meyer and Hostetter, 2007). Nevertheless, previous studies have argued that urea itself causes only a minor portion of uremic illness (Johnson et al., 1972). Thus, it is imperative to take efficient means to remove excess fluids and electrolytes, especially toxic metabolic wastes, or selectively remove uremic toxins that are known to cause specific symptoms, thus reducing the high morbidity and mortality of dialysis-dependent patients. Among them, PBUTs are hard to remove due to their attachment to the transport protein human serum albumin. Accumulating evidence has shown that it is not just downstream toxin removal that we need to focus on; reducing upstream toxin production can be a successful strategy by providing more approaches. The eliminations of uremic toxins are shown in Figure 2.

### 3.1 Reduction in the generation of gut-derived uremic toxins

New, emerging approaches have exhibited a distinctive effect on the elimination of uremic toxins of gut origin, which is accompanied by the evolution of various biotechnologies. Therefore, targeting the gut might be a promising adjuvant way to tackle stubborn uremic toxins.

The gut microbiome and CKDs share a complex bilateral relationship. (Mishima et al., 2017) designed a clever experiment that compared germ-free (GF) and specific pathogen-free (SPF) IQI mice and found that mice with renal failure housed under GF conditions had significantly lower levels of microbiota-derived uremic solutes in their plasma metabolites. In addition, compared to SPF mice with renal failure, mice with renal failure under GF conditions caused the disappearance of colonic short-chain fatty acids (SCFAs), a reduction in the utilization of intestinal amino acids, and more severe renal damage, which may further exacerbate renal damage in GF mice with renal failure. The authors thought that development without microbiota has detrimental effects on CKD progression and contributes significantly to the formation of toxic uremic solutes (Mishima et al., 2017). Additionally, a previous study demonstrated that it is possible to control host IS levels by targeting the microbiota and suggested a possible strategy for treating renal diseases (Devlin et al., 2016).

Bacterial metabolism plays a nonnegligible role in toxin generation. As Evenepoel et al. (Evenepoel et al., 2009) summarized, bacterial species can be roughly categorized as saccharolytic (i.e., those that predominantly ferment carbohydrates) or proteolytic (i.e., those that are predominantly protein fermenters) (Hida et al., 1996; Takayama et al., 2003; Taki et al., 2005; Goldfarb et al., 2007). It is widely acknowledged that the availability of nutrients, notably the ratio of available carbohydrates to nitrogen, which affects the degree of saccharolytic vs proteolytic fermentation, is the most significant regulator of bacterial metabolism (McOrist et al., 2011). Hence, the composition, dynamics, and stability of the gut microbiome could be a therapeutic target for removing uremic toxins (Ramezani and Raj, 2014b). Huang et al. developed a probiotic screening platform based on gut-derived uremic toxin-reducing probiotics and selected two strains (*Lactobacillus* paracasei and *Lactobacillus* plantarum) that display kidney protective functions. These bacteria act by reducing kidney injury and fibrotic-related proteins, decreasing oxidative stress and proinflammatory reactions and elevating immune responses, reversing gut dysbiosis, and restoring the abundance of commensal bacteria, especially SCFA producers, which leads to improved intestinal barrier integrity via modulation of the microbial composition and metabolite production (Huang et al., 2021a) (Figure 2C). By improving the available carbohydrates to nitrogen ratio, increasing the production of SCFAs, lowering the colonic pH, lengthening the colonic transit time, and suppressing the enzymes that catalyze the reactions that result in relative toxins, prebiotics and probiotics may help bring about such a change in the colonic environment (Rossi et al., 2012). Vaziri et al. demonstrated a significant difference in the abundance of 175 bacterial operational taxonomic units between uremic and normal rats, most notably observed as decreases in the Lactobacillaceae and Prevotellaceae families (Vaziri et al., 2013), which can be supplemented by probiotics. In PD patients receiving probiotics, the levels of serum cytokines and endotoxin significantly decreased after 6 months of treatment (Wang et al., 2015). For patients with stage 3–5 CKD, prebiotics significantly reduced the levels of uremic toxin of intestinal origin and favorably affected the gut microbiome (Ebrahim et al., 2022). By targeting one or a few healthy bacteria to promote their growth and/or activate their metabolism, synbiotics, a combination of prebiotics and probiotics, have been shown to have a positive effect on the host and improve the survival and implantation of live microbial dietary supplements in the gastrointestinal tract (Pandey et al., 2015).(Haghighat et al., 2020) designed a randomized double-blind controlled trial to explore the effects of probiotic and synbiotic supplementation in HD patients, and found that administration of synbiotics was more effective than probiotics for improving inflammatory markers, endotoxin and anti-HSP70 serum levels. The rise in the genetic engineering of bacteria reduces gut microbial-target uremic toxins as promising and potential candidates (Lim and Song, 2019). Similar to prebiotics, fecal microbiota transplantation (FMT) is a therapeutic method that targets the restoration of intestinal flora balance and curbing diseases stemming from such imbalances. This technique offers a fresh approach to eradicating uremic toxins. In clinical practice, FMT entails introducing fecal matter from healthy donors into the patient’s gut, allowing for the establishment of a robust and healthy microbiome. (Wang et al., 2020) conducted a comprehensive study that showed that a decrease of the driver gut bacterial species abundance should attenuate the severity of the disease, by transplanting the fresh gut microbiota from either patient with ESRD or healthy donors into germ-free CKD mice.

Numerous studies have demonstrated that dietary changes may reduce uremic toxin levels. A vegetable-based diet high in fiber appears to offer important advantages to CKD or dialysis-dependent patients (de Brito-Ashurst et al., 2009; Goraya et al., 2012; Kalantar-Zadeh et al., 2015; Rossi et al., 2015; Kandouz et al., 2016). A high-fiber, low-protein, plant-based diet may favorably alter the gut microbiota in a manner that reduces uremic toxin production and inhibits CKD progression (Kalantar-Zadeh et al., 2020). Additionally, very low protein diets (protein intake of 0.4 g/kg/day) were linked to better preservation of kidney function and a slower rate of end-stage renal disease (ESRD) development (Rhee et al., 2018). According to an 8-week randomized controlled trial that included 50 ESRD patients, supplementation of the diet with a high content of fermentable fiber decreased the serum levels of some nitrogenous products, such as serum creatinine and p-cresol, without changing the IS levels in maintenance HD patients (Khosroshahi et al., 2019). SCFAs, a fermentation end product with anti-inflammatory and histone deacetylase inhibiting properties produced by the intestinal microbiota, may modulate the inflammatory response and lessen the effects of hypoxia in kidney epithelial cells by enhancing mitochondrial biogenesis (Andrade-Oliveira et al., 2015). Supplementation with SCFAs, especially sodium propionate, could effectively lower the essential gut-derived uremic toxins indoxyl and p-cresol sulfate (Marzocco et al., 2018). Targeting the gut microbiome and making dietary changes are relatively safe methods without clear side effects that are suitable for maintenance dialysis patients.

### 3.2 Strengthening the removal of uremic toxins

#### 3.2.1 Peritoneal dialysis

PD is a widely used renal replacement therapy. It removes excess water and toxins via the peritoneum, a natural semipermeable membrane, and provides more flexibility and independence for patients (Figure 2B). The understanding of solute and water transport processes across the peritoneum underlies the efficiency of PD. The continuous capillary endothelium, peritoneal interstitial space, and mesothelium are pathways in which solutes and water can be exchanged between the plasma in the peritoneal capillaries and the fluid in the peritoneal cavity (Devuyst and Rippe, 2014). The water channel aquaporin-1 (AQP1) is constitutively produced in the endothelial cells lining peritoneal capillaries and allows osmotic water transfer across these barriers. In PD, where water is osmotically extracted into the peritoneal fluid by hyperosmolar dialysate solutions, AQP1 offers a water-only channel for fluid clearance (Verkman, 2006). To date, a few case‒control studies have found that HD is far more effective that PD in removing PBUTs (Yoshida et al., 2007; Pham et al., 2008), and automated PD also less effectively removes PBUTs than continuous ambulatory PD, an advantage (Eloot et al., 2015) that may be related to residual renal function in PD patients. A systematic review and meta-analysis showed that the only statistically significant difference between the quality of life of patients on HD and PD is in regards to the effects of kidney disease, which happens to be better in patients undergoing PD (Zazzeroni et al., 2017).

#### 3.2.2 Extracorporeal hemodialysis techniques

Therapeutic HD removes solutes primarily by adsorption, diffusion, and convection across semipermeable membranes (Figure 2A). The driving force for solute diffusion is the concentration gradient across the membrane, and for convection, generally referred to as ultrafiltration, the driving force is the transmembrane hydrostatic pressure. In recent years, how to effectively remove PBUTs from the serum of HD patients has become a research hotspot in the field of blood purification. Increasing the treatment time and frequency to prolong the HD time can only improve the clearance rate of small, water-soluble molecular solutes and medium and large molecular toxins (Basile et al., 2011). Long-term HD at night had little effect on improving the clearance of PBUTs (Meijers et al., 2011) and did not meet expectations. Initial experience suggested that hemofiltration (HF) and hemodiafiltration (HDF) may augment the removal of larger molecules and protein-bound solutes through increased convective clearance (Blankestijn et al., 2010; Eloot et al., 2012; Susantitaphong et al., 2013). Therefore, increasing the level of free PBUTs and extracorporeal adsorption are indispensable. PBUTs in the blood mostly bind reversibly to the Sudlow I or II site of serum albumin via noncovalent bonds, but this binding is affected by the temperature, pH, dilution factor of the blood and concentration of ions and drugs. Infusing ibuprofen, a binding competitor, into the arterial bloodline during HD significantly increases the dialytic removal of IS and pCS and thereby leads to a greater reduction in their serum levels (Madero et al., 2019). Moreover, high sodium concentrations in the substituate of predilution HDF at increased plasma ionic strength could improve PBUT removal (Krieter et al., 2017). In addition, some studies have found that the use of high dissolved hydrogen dialysate can promote the dissociation of IS from the albumin binding site, increase the level of free IS and improving the scavenging effect of IS in the filtrate (Tange et al., 2015).

Combining extracorporeal HD with the adsorption technique seems to be another trend in removing PBUTs. A hemoperfusion cartridge containing an adsorbent substance, such as activated charcoal or a resin, is used in this procedure to circulate blood extracorporeally. Adsorbents are substances that, as a result of their physical and chemical properties, adsorb other elements that dissolve on their surface (Botella et al., 2000). Almost all solutes in plasma are protein-bound to some extent (Keller et al., 1983). Additionally, the toxins that are protein-bound are not eliminated by HD, and the plasma proteins that bind drugs are too large to be removed via extracorporeal therapies other than apheresis (Lam et al., 1997; Roberts and Buckley, 2007). The molecular mass of substances amenable to extracorporeal removal has increased since the introduction of HP, which has improved to clear greater amounts of uremic toxins, especially middle molecules. In HD, conventional dialyzers may clear substances up to 15,000 Da, whereas high cutoff hemofilters may clear substances closer to 50,000 Da in size (Gondouin and Hutchison, 2011; Kirsch et al., 2017). Sorbents were first used in nephrology by Muirhead and Reid, who utilized an ionic resin to remove uremic toxins from dogs in 1948 (Muirhead and Reid, 1948). In recent years, there is a significant focus on researching, developing, and testing new adsorption materials. The bottom-up assembled adsorbents of the intrinsically biocompatible protein cage ferritin have been developed lately. Through the introduction of chemical modification to selectively target the inner surface, coupled with the functionalization of hydrophobic molecules, the level of adsorbed uremic toxins is greatly increased (Böhler et al., 2022). Traditional adsorption membrane materials are generally made of a mixture of traditional polymer materials and adsorption particles. The polymer matrix of the membrane is mostly polymer polyethersulfone/polyvinylpyrrolidone (PES/PVP), which is blended with various new nanomaterials to adsorb PBUTs. Moreover, metal-organic frameworks (MOFs) have shown promising performance in the adsorption of PBUTs, a class of crystalline micro/mesoporous hybrid materials composed of metal ions or metal clusters interconnected by organic linkers. An ultramicroporous olefin-linked COF (NKCOF-12) with good biocompatibility and excellent adsorption was successfully prepared via the melt polymerization synthesis method by Wei and his colleagues (Wei et al., 2023). At present, this kind of new composite materials with micro-nano-scale metal framework and organic ligands is a new direction of adsorbent research because of its superior adsorption performance, high specific surface area, and good biocompatibility. A pilot clinical trial in Vietnam showed that a combination of HD and hemoperfusion with HA 130 resin for 3 years helped to reduce the cardiovascular-related mortality rate (Nguyen Huu et al., 2021)^.^ Additionally, in a randomized control trial, patients who received polymyxin B hemoperfusion displayed significant decreases in cytokines after 3 days compared to those who received only standard treatment (Srisawat et al., 2018), and some researchers believe that prolonged polymyxin therapy might be associated with better clinical outcomes in patients with septic shock (Kawazoe et al., 2018). Currently, there is an efflux of newly designed materials with higher absorption rates and limited albumin removal. Magnani and Att, 2021, summarized the state of the art blood purification strategies and showed that adsorption-based extracorporeal techniques, particularly HDF with endogenous infusion and hemoperfusion, integrated directly with current HD systems that adsorbed large amounts of middle molecular weight molecules and PBUTs (Magnani and Atti, 2021). Apart from traditional charcoal, many resin sorbents, mainly those that are cellulosic or polymeric, demonstrate a more efficient rate of clearance (Yamamoto et al., 2018; Gemelli et al., 2019; Rocchetti et al., 2020). (Liu et al., 2021) prepared new biosafe and efficient nitrogen-containing porous carbon adsorbent (NPCA) beads for the clearance of PBUTs, the removal mechanism of which is ascribed to effective competition between the nitrogen-containing NPCA and proteins for PBUT binding. Shen et al., 2020. indicated that the dialysate supported by cationic liposomes significantly improved the efficiency of removing certain PBUTs, which suggests the potential of this cationic absorbent as an ideal scavenger for PBUTs in blood purification (Shen et al., 2020). Nevertheless, the use of hemoperfusion has declined to approximately 1% of HD utilization in the United States and has been traditionally used primarily for poisoning (Ghannoum et al., 2016) including paraquat poisoning (largely in Asia, and often in combination with HD) (Ouellet et al., 2014; Li et al., 2018).

HDF with sorbent-regenerated endogenous ultrafiltrate reinfusion (HFR) is a type of HDF in which the replacement fluid consists of the patient’s ultrafiltrate that has been regenerated through a cartridge with hydrophobic styrene resin. HFR may provide a favorable compromise between the optimization of toxin removal and the possible loss of beneficial physiological substances (Aucella, 2012). According to clinical research, HFR is associated with a better physical component in terms of health-related quality of life than bicarbonate HD that is independent of age, sex, dialysis vintage and invalidity score (Borrelli et al., 2016). In addition, HFR provides new treatment ideas for other diseases, such as multiple myeloma and acute kidney injury.

Hemoperfusion (HP) is based on the mass separation of adsorbents. A blood purification technique that introduces the patient’s blood into a perfusion device equipped with a solid adsorbent and removes exogenous or endogenous toxins, drugs, or metabolic waste from the blood that cannot be cleared by dialysis through adsorption. HD can only remove substances with high diffusion, non-protein binding, and small to medium molecular weight. The combination therapy of HD and HP improves the detoxification potential of chronic uremia, and is superior to HD in regularly clearing the accumulation of medium and large-molecule uremic toxins in the body (Splendiani et al., 1987). HD + HP has a potential role in improving patients’ quality of life and survival rate. HDF combined with HP treatment mainly removes middle molecular substances, while the latter ensures that a large amount of toxin metabolites are cleared through diffusion and convection. The combination of the two treatments can exert synergistic clearance and adsorption effects, prevent further damage to the patient’s kidney function, control the prognosis of the condition, and improve the patient’s physical symptoms (Fan, 2021).

#### 3.2.3 Gut-target toxin removal techniques

Intraluminal adsorption onto high-affinity surfaces may also serve as an effective intervention for toxin removal. An oral preparation of activated charcoal has demonstrated its effectiveness in toxin removal (Botella et al., 2000; Cupisti et al., 2020). AST-120, prescribed to CKD patients for over 2 decades, is an orally available intestinal adsorbent composed of porous carbon particles that are 0.2–0.4 mm in diameter (Niwa et al., 1991; Schulman et al., 2014) (Figure 2D). A systematic review produced conclusive evidence on the effectiveness of AST-120 in delaying the progression of CKD (Su et al., 2021). (Sato et al., 2017) concluded that uremic toxins can accumulate in a variety of organs and that AST-120 may be useful in the treatment or prevention of organ dysfunction in CKD, possibly by reducing tissue accumulation of uremic toxins. In detail, AST-120 has the capacity to bind to the precursor of IS in the intestinal tract, efficiently suppress IS production in the liver (Nagata and Yoshizawa, 2020), effectively reduce serum and hippocampal IS levels and reverse cognitive impairment in CKD mice (Li et al., 2021a). Furthermore, in diabetic mice, AST-120 reduced the serum levels of AGEs and enhanced the neovascularization of ischemic limbs, which may be due to changes in macrophage polarization and the corresponding shifts in inflammatory cytokines (Huang et al., 2021b), and prevented the decrease in soluble Flt-1 expression, which is an endogenous antagonist of atherosclerotic progression in CKD (Nakada et al., 2019). Similarly, AST-120 was proven in a clinical trial to ameliorate microvascular endothelial dysfunction and carotid arterial intima-media thickness in HD patients (Ryu et al., 2016). This may be because AST-120 decreases the generation of reactive oxygen species by endothelial cells to impede ensuing oxidative stress (Liu et al., 2018; Hwang et al., 2019). Along with its effect on cardiovascular disease, AST-120 may alleviate inflammation and oxidative stress in primary central nervous system cells and IS-induced neuronal death through activation of nuclear factor-κB (NF-κB) and aryl hydrocarbon receptors (Adesso et al., 2018).

The use of intestinal phosphate binders is a proven and effective method to lessen the load of phosphorus on the kidneys to limit the increased risk of CKD-mineral and bone disorder (MBD) and its associated morbidity and mortality as well as stop the illness from progressing (National Kidney Foundation, 2003; Malberti, 2013). However, considering the difficulty of constantly maintaining phosphate-restricted diets, it is more practical to pharmacologically target the intestinal fractional absorptive capacity by blocking phosphate transporters or altering the intestinal epithelium tight junctions so that they are less permeable to phosphate ions (Yu et al., 2010; Stremke and Hill Gallant, 2018). Phosphate binders fall into two categories: 1) calcium-based binders (calcium carbonate, calcium acetate, and calcium acetate/magnesium carbonate) and 2) noncalcium-based binders (sevelamer, lanthanum, and, more recently, iron-based binders) (Laville et al., 2021). Researchers discovered in a randomized controlled experiment that phosphate binders effectively reduced serum and urine phosphorus and slowed the evolution of secondary hyperparathyroidism in CKD patients with normal or nearly normal levels of serum phosphorus. However, phosphate binders, including calcium acetate, lanthanum carbonate and sevelamer carbonate, also promote the progression of vascular calcification (Block et al., 2012). That is, the researchers believed that the safety and efficacy of phosphate binders in CKD remain uncertain (Block et al., 2012). Another adverse view came from an Australian research team who concluded that treating patients with stage 3b/4 CKD with lanthanum for 96 weeks had no effect compared to placebo in terms of reducing arterial stiffness or aortic calcification. These results refute the idea that intestinal phosphate binders can lower cardiovascular risk in CKD patients with normophosphatemia (Toussaint et al., 2020). Repositioned phosphate binders do not seem to be able to significantly lower circulating levels of these toxic substances, although they impede the absorption of both phosphate and gut-derived uremic toxins (Laville et al., 2021) and reduce toxin damage to the body.

Colonic dialysis has long been used in China to help remove gut-derived toxins to delay CKD progression. Colon dialysis involves injecting filtered water into the human colon to clean the colon, remove toxins from the body, and fully expand the contact area between the colonic mucosa and the drug. Then, a special medicinal liquid is injected for its absorption by the colon through the colonic mucosa and out of the body. Toxins can be discharged over time and finally reinfused with special traditional Chinese medicine preparations and retained. The colonic mucosa is used to absorb the active ingredients drugs in the colon, producing a therapeutic effect on the kidneys and reducing reflux, turbidity and serum uremic toxins. Colonic dialysis could significantly improve the richness of the gut microbiome, bringing it closer to the profile in healthy subjects (Li et al., 2021b), acting as an effective supplementary therapy to delay the progression of stage 4–5 CKD (Dai et al., 2019). With the Chinese herbal formula Gubenxiezhuo, colonic dialysis could significantly ameliorate inflammation to modulate the distribution of the gut microbiota in uremia (He et al., 2019).

#### 3.2.4 Natural products as a therapeutic approach

Natural products are usually identified as chemical substances produced by living organisms that can be found in nature and have distinct pharmacological effects. Natural products have been considered a promising and effective treatment for ESRD, as they may facilitate the removal of uremic toxins and reduce the damage uremic toxins cause to the human body (Figure 2E).

The intestinal mucosal barrier and gut microbiome may be prospective therapeutic targets against the progression of CKD (Yang et al., 2019). Modern pharmacological studies have shown that rhubarb contains free anthraquinone derivatives such as rhein, emodin, chrysophanol, aloeemodin, and emodin-3-methyl ether. Animal experiments revealed that rhubarb enema treatment could decrease serum levels of IS, renal oxidative stress, and NF-κB levels, attenuate histopathological changes, and restore the intestinal mucosal barrier by modifying several functional enteric bacteria, which may be associated with reduced inflammation and ameliorated kidney tubulointerstitial fibrosis (Lu et al., 2015; Ji et al., 2020). Supporting data have shown that α-ketoacid significantly improves the intestinal barrier and affects the intestinal microbial community, showing a renoprotective effect against adenine-induced CKD (Mo et al., 2021). In addition, resveratrol or resveratrol butyrate ester have been proposed to shape the gut microbiota composition and target the gut–kidney axis to prevent adenine-induced kidney injury and hypertension. This may be related to reduced renal expression of SCFA G protein-coupled receptor 41 and olfactory receptor 78, antagonizing the AhR signaling pathway, and the increased abundance of beneficial bacteria such as the genera Akkermansia, Blautia, and *Enterococcus* (Hsu et al., 2021). The traditional Chinese medicine formulation Qiong-Yu-Gao, which is made from the radices of Rehmanniae, Poria, and ginseng, significantly reduced gut dysbiosis, changed the levels of bacterial metabolites—increasing SCFAs such as acetic acid and butyric acid and decreasing uremic toxins such as IS and pCS—and suppressed histone deacetylase expression and activity (Zou et al., 2022).

Beyond the kidney–gut function axis, natural products exhibit various abilities to inhibit CKD progression by removing uremic toxins. Li et al., 2021a, proposed that the use of Danhong injection or salvianolic acids as protein-bound competitors is superior to previously reported strategies and drugs for the removal of the PBUTs IS and pCS (Li et al., 2019). Many convincing data have been obtained from a number of clinical trials and animal experiments demonstrating that natural products exert their nephroprotective effects via diverse signaling pathways, especially the TGF-β pathway, thus increasing exchange transport and decreasing oxidative stress and apoptosis to facilitate the excretion of uremic toxins (Wang et al., 2018; Wang et al., 2019; Ishimitsu et al., 2021; Yu et al., 2021; Zheng et al., 2021; Liu et al., 2022; Ren et al., 2022).

Numerous natural products contain intricate active ingredients that target various biological factors, rendering them a promising option for individuals suffering from CKD. This method is less invasive and simpler than conventional toxin removal approaches. However, despite the advantages, natural products are not yet extensively used due to the challenge of identifying and researching the mechanisms of their active ingredients. This area of study will be a critical focus of future research.

## 4 Summary

Uremic toxins have deleterious effects on the human body and managing the retained solutes that are poorly eliminated by the applied treatment remains a difficult challenge. Although complicated composition of uremic toxins is being unraveled largely using mass spectrometry, there is a need for experiments using mouse models, cells system and physiologically relevant experimental conditions combined with questionnaire survey. PBUTs are notoriously difficult to remove, however, in order to reduce its accumulation and toxicity, the question remains whether to reduce production or promote its free state in the circulation. The need for precise and accurate characterization of uremic toxins will continue to grow as our knowledge of their amount, accumulation, and toxicity evolves. It remains a knotty problem that matching a single toxin or a group of toxins to a symptom. The field is in urgent need of animal models and organoid experiments with which to study accumulation, removal, and toxicity of uremic toxins. The rapid development of medical technology focused on toxin removal has hindered toxicological research to a certain extent, and more research is needed for a better understanding of uremic solutes and their toxic effects. It has been noted that the gut-kidney axis has provided valuable insights in the study of kidney disease and uremic toxins. In light of this, researchers and medical professionals may consider exploring potential avenues for targeting the gut in efforts to eliminate these toxins in the future. This will make dialysis more rational and should lead to more effective treatments.

## References

[B1] Abordo E. A. Minhas H. S. Thornalley P. J. (1999). Accumulation of alpha-oxoaldehydes during oxidative stress: a role in cytotoxicity. Biochem. Pharmacol. 58 (4), 641–648. 10.1016/s0006-2952(99)00132-x 10413301

[B3] Adelibieke Y. Yisireyili M. Ng H. Y. Saito S. Nishijima F. Niwa T. (2014). Indoxyl sulfate induces IL-6 expression in vascular endothelial and smooth muscle cells through OAT3-mediated uptake and activation of AhR/NF-κB pathway. Nephron Exp. Nephrol. 128 (1–8), 1–8. 10.1159/000365217 25376195

[B4] Adesso S. Paterniti I. Cuzzocrea S. Fujioka M. Autore G. Magnus T. (2018). AST-120 reduces neuroinflammation induced by indoxyl sulfate in glial cells. J. Clin. Med. 7 (10), 365. 10.3390/jcm7100365 30336612 PMC6210605

[B5] Adijiang A. Higuchi Y. Nishijima F. Shimizu H. Niwa T. (2010). Indoxyl sulfate, a uremic toxin, promotes cell senescence in aorta of hypertensive rats. Biochem. Biophys. Res. Commun. 399, 637–641. 10.1016/j.bbrc.2010.07.130 20691162

[B6] Agus A. Planchais J. Sokol H. (2018). Gut microbiota regulation of tryptophan metabolism in health and disease. Cell. Host Mi-crobe 23 (6), 716–724. 10.1016/j.chom.2018.05.003 29902437

[B7] Almeras C. Argilés A. (2009). The general picture of uremia. Semin. Dial. 22 (4), 329–333. 10.1111/j.1525-139X.2009.00575.x 19708976

[B8] Andrade-Oliveira V. Amano M. T. Correa-Costa M. Castoldi A. Felizardo R. J. F. de Almeida D. C. (2015). Gut bacteria products prevent AKI induced by ischemia-reperfusion. J. Am. Soc. Nephrol. JASN 26 (8), 1877–1888. 10.1681/ASN.2014030288 25589612 PMC4520159

[B9] Aucella F. (2012). Emodiafiltrazione con reinfusione endogena (HFR) (Hemodiafiltration with endogenous reinfusion). G. Ital. Nefrol. 29 (Suppl. 55), S72–S82. 22723147

[B10] Banoglu E. Jha G. G. King R. S. (2001). Hepatic microsomal metabolism of indole to indoxyl, a precursor of indoxyl sulfate. Eur. J. Drug Metab. Pharmacokinet. 26 (4), 235–240. 10.1007/BF03226377 11808865 PMC2254176

[B11] Barreto F. C. Barreto D. V. Liabeuf S. Meert N. Glorieux G. Temmar M. (2009). Serum indoxyl sulfate is associated with vascular disease and mortality in chronic kidney disease patients. Clin. J. Am. Soc. Nephrol. 4, 1551–1558. 10.2215/CJN.03980609 19696217 PMC2758258

[B12] Basile C. Libutti P. Di Turo A. L. Casino F. G. Vernaglione L. Tundo S. (2011). Removal of uraemic retention solutes in standard bicarbonate haemodialysis and long-hour slow-flow bicarbonate haemodialysis. Nephrol. Dial. Transpl. 26 (4), 1296–1303. 10.1093/ndt/gfq543 20813765

[B13] Blankestijn P. J. Ledebo I. Canaud B. (2010). Hemodiafiltration: clinical evidence and remaining questions. Kidney Int. 77 (7), 581–587. 10.1038/ki.2009.541 20130529

[B14] Block G. A. Wheeler D. C. Persky M. S. (2012). Effects of phosphate binders in moderate CKD. J. Am. Soc. Nephrol. 23 (8), 1407–1415. 10.1681/ASN.2012030223 22822075 PMC3402292

[B15] Böhler H. Orth-Alampour S. Baaten C. Riedner M. Jankowski J. Beck T. (2022). Assembly of chemically modified protein nanocages into 3D materials for the adsorption of uremic toxins. J. Mater Chem. B 11 (1), 55–60. 10.1039/d2tb02386e 36504125

[B16] Borrelli S. Minutolo R. De Nicola L. De Simone W. De Simone E. Zito B. (2016). Quality of life of hemodialysis patients in Central and Southern Italy: cross-sectional comparison between Hemodiafiltration with endogenous reinfusion (HFR) and Bicarbonate Hemodialysis. Qualità della vita dei pazienti in emodialisi nel centro-sud Italia: confronto tra Emodiafiltrazione a reinfusione endogena (HFR) e Bicarbonato Dialisi. G. Ital. Nefrol. 33 (3), gin/33.3.8. 27374393

[B17] Botella J. Ghezzi P. M. Sanz-Moreno C. (2000). Adsorption in hemodialysis. Kidney Int. Suppl. 76, S60–S65. 10.1046/j.1523-1755.2000.07607.x 10936800

[B18] Brunelli L. Davin A. Sestito G. (2021). Plasmatic hippuric acid as a hallmark of frailty in an Italian cohort: the mediation effect of fruit-vegetable intake. J. Gerontol. A Biol. Sci. Med. Sci. 76 (12), 2081–2089. 10.1093/gerona/glab244 34436596 PMC8599087

[B19] Chen L. Wang D. Garmaeva S. (2021). The long-term genetic stability and individual specificity of the human gut microbiome. Cell. 184 (9), 2302–2315.e12. 10.1016/j.cell.2021.03.024 33838112

[B20] Chmielewski M. Cohen G. Wiecek A. Jesús Carrero J. (2014). The peptidic middle molecules: is molecular weight doing the trick? Semin. Nephrol. 34 (2), 118–134. 10.1016/j.semnephrol.2014.02.005 24780468

[B21] Clark W. R. Dehghani N. L. Narsimhan V. Ronco C. (2019). Uremic toxins and their relation to dialysis efficacy. Blood Purif. 48 (4), 299–314. 10.1159/000502331 31563911

[B22] Cupisti A. Piccoli G. B. Gallieni M. (2020). Charcoal for the management of pruritus and uremic toxins in patients with chronic kidney disease. Curr. Opin. Nephrol. Hypertens. 29 (1), 71–79. 10.1097/MNH.0000000000000567 31725009

[B23] Dai S. Dai Y. Peng J. Xie X. Ning J. (2019). Simplified colonic dialysis with hemodialysis solutions delays the progression of chronic kidney disease. Qjm 112 (3), 189–196. 10.1093/qjmed/hcy260 30407603

[B24] de Brito-Ashurst I. Varagunam M. Raftery M. J. Yaqoob M. M. (2009). Bicarbonate supplementation slows progression of CKD and improves nutritional status. J. Am. Soc. Nephrol. 20 (9), 2075–2084. 10.1681/ASN.2008111205 19608703 PMC2736774

[B25] De Simone G. Balducci C. Forloni G. Pastorelli R. Brunelli L. (2021). Hippuric acid: could became a barometer for frailty and geriatric syndromes? Ageing Res. Rev. 72, 101466. 10.1016/j.arr.2021.101466 34560280

[B26] Devlin A. S. Marcobal A. Dodd D. Nayfach S. Plummer N. Meyer T. (2016). Modulation of a circulating uremic solute via rational genetic manipulation of the gut microbiota. Cell. Host Microbe 20 (6), 709–715. 10.1016/j.chom.2016.10.021 27916477 PMC5159218

[B27] Devuyst O. Rippe B. (2014). Water transport across the peritoneal membrane. Kidney Int. 85 (4), 750–758. 10.1038/ki.2013.250 23802191

[B28] Dou L. Bertrand E. Cerini C. Faure V. Sampol J. Vanholder R. (2004). The uremic solutes p-cresol and indoxyl sulfate inhibit endothelial proliferation and wound repair. Kidney Int. 65, 442–451. 10.1111/j.1523-1755.2004.00399.x 14717914

[B29] Dou L. Cerini C. Brunet P. Guilianelli C. Moal V. Grau G. 2002. P-cresol, a uremic toxin, decreases endothelial cell response to inflammatory cytokines. Kidney Int. 62: 1999, 2009. 10.1046/j.1523-1755.2002.t01-1-00651.x 12427124

[B30] Duncan S. H. Richardson A. J. Kaul P. Holmes R. P. Allison M. J. Stewart C. S. (2002). Oxalobacter formigenes and its potential role in human health. Appl. Environ. Microbiol. 68 (8), 3841–3847. 10.1128/aem.68.8.3841-3847.2002 12147479 PMC124017

[B31] Duranton F. Cohen G. De Smet R. (2012). Normal and pathologic concentrations of uremic toxins. J. Am. Soc. Nephrol. 23 (7), 1258–1270. 10.1681/ASN.2011121175 22626821 PMC3380651

[B32] Ebrahim Z. Proost S. Tito R. Y. (2022). The effect of ß-glucan prebiotic on kidney function, uremic toxins and gut microbiome in stage 3 to 5 chronic kidney disease (CKD) predialysis participants: a randomized controlled trial. Nutrients 14 (4), 805. 10.3390/nu14040805 35215453 PMC8880761

[B33] Eloot S. Van Biesen W. Vanholder R. (2012). A sad but forgotten truth: the story of slow-moving solutes in fast hemodialysis. Semin. Dial. 25 (5), 505–509. 10.1111/j.1525-139X.2012.01107.x 22925227

[B34] Eloot S. Vanholder R. Dequidt C. Van Biesen W. (2015). Removal of different classes of uremic toxins in apd vs capd: a ran-domized cross-over study. Perit. Dial. Int. 35 (4), 436–442. 10.3747/pdi.2013.00202 24584609 PMC4520726

[B35] Evenepoel P. Meijers B. K. I. Bammens B. R. M. Verbeke K. (2009). Uremic toxins originating from colonic microbial metabolism. Kidney Int. 76, S12–S19. 10.1038/ki.2009.402 19946322

[B36] Fan C. M. (2021). Effect of hemodiafiltration combined with hemoperfusion in the treatment ofuremia and its effect on biochemical indices. J. Nongken Med. 43 (04), 299–302.

[B37] Fan Y. Pedersen O. (2021). Gut microbiota in human metabolic health and disease. Nat. Rev. Microbiol. 19 (1), 55–71. 10.1038/s41579-020-0433-9 32887946

[B38] Gao Y. Zhang J. Chen H. Wang Z. Hou J. Wang L. (2021). Dimethylamine enhances platelet hyperactivity in chronic kidney disease model. J. Bioenerg. Biomembr. 53 (5), 585–595. 10.1007/s10863-021-09913-4 34327565

[B39] Gemelli C. Cuoghi A. Magnani S. Atti M. Ricci D. Siniscalchi A. (2019). Removal of bilirubin with a new adsorbent system: *in vitro* kinetics. Blood Purif. 47 (1-3), 10–15. 10.1159/000492378 30219813

[B40] Ghannoum M. Lavergne V. Gosselin S. Mowry J. B. Hoegberg L. C. G. Yarema M. (2016). Practice trends in the use of extracorporeal treatments for poisoning in four countries. Semin. Dial. 29 (1), 71–80. 10.1111/sdi.12448 26551956

[B41] Glassock R. J. Massry S. G. (2022). “Chapter 6 - uremic toxins: an integrated overview of classification and pathobiology,” in Nutritional management of renal disease Kopple J. D. Massry S. G. Kalantar-Zadeh K. Fouque D. Fourth Edition (China: Academic Press), 77–89.

[B42] Goldfarb D. S. Modersitzki F. Asplin J. R. (2007). A randomized, controlled trial of lactic acid bacteria for idiopathic hyperoxaluria. Clin. J. Am. Soc. Nephrol. 2 (4), 745–749. 10.2215/CJN.00600207 17699491

[B43] Gondouin B. Hutchison C. A. (2011). High cut-off dialysis membranes: current uses and future potential. Adv. Chronic Kidney Dis. 18 (3), 180–187. 10.1053/j.ackd.2011.02.006 21531324

[B44] Goraya N. Simoni J. Jo C. Wesson D. E. (2012). Dietary acid reduction with fruits and vegetables or bicarbonate attenuates kidney injury in patients with a moderately reduced glomerular filtration rate due to hypertensive nephropathy. Kidney Int. 81 (1), 86–93. 10.1038/ki.2011.313 21881553

[B45] Gouroju S. Rao P. V. L. N. S. Bitla A. R. Vinapamula K. S. Manohar S. M. Vishnubhotla S. (2017). Role of gut-derived uremic toxins on oxidative stress and inflammation in patients with chronic kidney disease. Indian J. Nephrol. 27 (5), 359–364. 10.4103/ijn.IJN_71_17 28904431 PMC5590412

[B46] Graboski A. L. Redinbo M. R. (2020). Gut-derived protein-bound uremic toxins. Toxins 12 (9), 590. 10.3390/toxins12090590 32932981 PMC7551879

[B47] Gryp T. De Paepe K. Vanholder R. Kerckhof F. M. Van Biesen W. Van de Wiele T. (2020). Gut microbiota generation of protein-bound uremic toxins and related metabolites is not altered at different stages of chronic kidney disease. Kidney Int. 97 (6), 1230–1242. 10.1016/j.kint.2020.01.028 32317112

[B48] Gryp T. Vanholder R. Vaneechoutte M. Glorieux G. (2017). p-Cresyl Sulfate. Toxins (Basel) 9 (2), 52. 10.3390/toxins9020052 28146081 PMC5331431

[B49] Haghighat N. Mohammadshahi M. Shayanpour S. Haghighizadeh M. H. (2020). Effects of synbiotics and probiotics supplementation on serum levels of endotoxin, heat shock protein 70 antibodies and inflammatory markers in hemodialysis patients: A randomized double-blinded controlled trial. Probiotics Antimicrob. Proteins 12 (1), 144–151. 10.1007/s12602-018-9509-5 30617950

[B50] He H. Hu P. Tang Y. Xu X. (2019). Influence of colonic dialysis using Gubenxiezhuo on the distribution of gut microflora in uremia rats. J. Cell. Physiol. 234 (7), 11882–11887. 10.1002/jcp.27845 30536550

[B51] Hida M. Aiba Y. Sawamura S. Suzuki N. Satoh T. Koga Y. (1996). Inhibition of the accumulation of uremic toxins in the blood and their precursors in the feces after oral administration of Lebenin®, a lactic acid bacteria preparation, to uremic patients undergoing hemodialysis. Nephron 74 (2), 349–355. 10.1159/000189334 8893154

[B52] Hill M. J. (1997). Intestinal flora and endogenous vitamin synthesis. Eur. J. Cancer Prev. 6 (Suppl. 1), S43–S45. 10.1097/00008469-199703001-00009 9167138

[B158] Himmelfarb J. Sayegh M. H. (Editors) (2011). Chronic kidney disease, dialysis, and transplantation : companion to Brenner & Rector’s the kidney. 3rd Edn. Philadelphia: Saunders, 251–264.

[B53] Hooper L. V. Midtvedt T. Gordon J. I. (2002). How host-microbial interactions shape the nutrient environment of the mammalian intestine. Annu. Rev. Nutr. 22, 283–307. 10.1146/annurev.nutr.22.011602.092259 12055347

[B54] Hsu C-N. Hou C-Y. Chang C-I. Tain Y-L. (2021). Resveratrol butyrate ester protects adenine-treated rats against hypertension and kidney disease by regulating the gut-kidney Axis. Antioxidants (Basel). 11 (1), 83. 10.3390/antiox11010083 35052587 PMC8772985

[B55] Huang H. Li K. Lee Y. Chen M. (2021a). Preventive effects of Lactobacillus mixture against chronic kidney disease progression through enhancement of beneficial bacteria and downregulation of gut-derived uremic toxins. J. Agric. Food Chem. 69 (26), 7353–7366. 10.1021/acs.jafc.1c01547 34170659

[B56] Huang H. L. Kuo C. S. Chang T. Y. (2021b). An oral absorbent, AST-120, restores vascular growth and blood flow in ischemic muscles in diabetic mice via modulation of macrophage transition. J. Mol. Cell. Cardiol. 155, 99–110. 10.1016/j.yjmcc.2021.03.001 33713645

[B57] Hwang Y. C. Kim S. W. Hur K. Y. Cha B. S. Kim I. J. Park T. S. (2019). Predictive factors for efficacy of AST-120 treatment in diabetic nephropathy: a pro-spective single-arm, open-label, multi-center study. J. Korean Med. Sci. 34 (15), e117. 10.3346/jkms.2019.34.e117 31001934 PMC6473095

[B58] Hylemon P. B. Harder J. (1998). Biotransformation of monoterpenes, bile acids, and other isoprenoids in anaerobic ecosystems. FEMS Microbiol. Rev. 22 (5), 475–488. 10.1111/j.1574-6976.1998.tb00382.x 9990726

[B59] Ishimitsu A. Tojo A. Satonaka H. Ishimitsu T. (2021). Eucommia ulmoides (Tochu) and its extract geniposidic acid reduced blood pressure and improved renal hemodynamics. Biomed. Pharmacother. 141, 111901. 10.1016/j.biopha.2021.111901 34328117

[B60] Itoh Y. Ezawa A. Kikuchi K. Tsuruta Y. Niwa T. (2012). Protein-bound uremic toxins in hemodialysis patients measured by liquid chromatography/tandem mass spectrometry and their effects on endothelial ROS production. Anal. Bioanal. Chem. 403 (7), 1841–1850. 10.1007/s00216-012-5929-3 22447217

[B61] Ji C. Deng Y. Yang A. (2020). Rhubarb enema improved colon mucosal barrier injury in 5/6 nephrectomy rats may associate with gut microbiota modification. Front. Pharmacol. 11, 1092. 10.3389/fphar.2020.01092 32848732 PMC7403201

[B62] Johnson W. J. Hagge W. W. Wagoner R. D. Dinapoli R. P. Rosevear J. W. (1972). Effects of urea loading in patients with far-advanced renal failure. Mayo Clin. Proc. 47 (1), 21–29. 5008253

[B63] Kalantar-Zadeh K. Tortorici A. R. Chen J. L. Kamgar M. Lau W. L. Moradi H. (2015). Dietary restrictions in dialysis patients: is there anything left to eat? Semin. Dial. 28 (2), 159–168. 10.1111/sdi.12348 25649719 PMC4385746

[B64] Kalantar-Zadeh K. Joshi S. Schlueter R. Cooke J. Brown-Tortorici A. Donnelly M. (2020). Plant-dominant low-protein diet for conservative management of chronic kidney disease. Nutrients 12 (7), 1931. 10.3390/nu12071931 32610641 PMC7400005

[B65] Kandouz S. Mohamed A. S. Zheng Y. Sandeman S. Davenport A. (2016). Reduced protein bound uraemic toxins in vegetarian kidney failure patients treated by haemodiafiltration. Hemodial. Int. 20 (4), 610–617. 10.1111/hdi.12414 27044443

[B66] Kawazoe Y. Sato T. Miyagawa N. Yokokawa Y. Kushimoto S. Miyamoto K. (2018). Mortality effects of prolonged hemoperfusion therapy using a polymyxin B-immobilized fiber column for patients with septic shock: a sub-analysis of the desire trial. Blood Purif. 46 (4), 309–314. 10.1159/000491744 30099437

[B67] Keller F. Wilms H. Schultze G. Offerman G. Molzahn M. (1983). Effect of plasma protein binding, volume of distribution and molecular weight on the fraction of drugs eliminated by hemodialysis. Clin. Nephrol. 19 (4), 201–205. 6851257

[B68] Khosroshahi H. T. Abedi B. Ghojazadeh M. Samadi A. Jouyban A. (2019). Effects of fermentable high fiber diet supplementation on gut derived and conventional nitrogenous product in patients on maintenance hemodialysis: a randomized controlled trial. Nutr. Metab. (Lond) 16, 18. 10.1186/s12986-019-0343-x 30911321 PMC6417142

[B69] Kirsch A. H. Lyko R. Nilsson L. G. Beck W. Amdahl M. Lechner P. (2017). Performance of hemodialysis with novel medium cut-off dialyzers. Nephrol. Dial. Transpl. 32 (1), 165–172. 10.1093/ndt/gfw310 PMC5837492 27587605

[B70] Krieter D. H. Devine E. Körner T. Rüth M. Wanner C. Raine M. (2017). Haemodiafiltration at increased plasma ionic strength for improved protein-bound toxin removal. Acta Physiol. (Oxf). 219 (2), 510–520. 10.1111/apha.12730 27259463

[B71] Lam Y. W. Banerji S. Hatfield C. Talbert R. L. (1997). Principles of drug administration in renal insufficiency. Clin. Pharmacokinet. 32 (1), 30–57. 10.2165/00003088-199732010-00002 9012555

[B72] Laville S. M. Massy Z. A. Kamel S. Chillon J. M. Choukroun G. Liabeuf S. (2021). Intestinal chelators, sorbants, and gut-derived uremic toxins. Toxins 13 (2), 91. 10.3390/toxins13020091 33530404 PMC7911578

[B73] Li C. Hu D. Xue W. Li X. Wang Z. Ai Z. (2018). Treatment outcome of combined continuous venovenous hemofiltration and hemoperfusion in acute paraquat poisoning: a prospective controlled trial. Crit. Care Med. 46 (1), 100–107. 10.1097/CCM.0000000000002826 29116999

[B74] Li J. Wang Y. Xu X. (2019). Improved dialysis removal of protein-bound uremic toxins by salvianolic acids. Phytomedicine 57, 166–173. 10.1016/j.phymed.2018.12.018 30772752

[B75] Li L. C. Chen W. Y. Chen J. B. (2021a). The AST-120 recovers uremic toxin-induced cognitive deficit via NLRP3 inflammasome pathway in astrocytes and microglia. Biomedicines 9 (9), 1252. 10.3390/biomedicines9091252 34572437 PMC8467651

[B76] Li Y. Dai M. Yan J. (2021b). Colonic dialysis can influence gut flora to protect renal function in patients with pre-dialysis chronic kidney disease. Sci. Rep. 11 (1), 12773. 10.1038/s41598-021-91722-1 34140540 PMC8211730

[B77] Liabeuf S. Barreto D. V. Barreto F. C. Meert N. Glorieux G. Schepers E. (2010). Free p-cresylsulphate is a predictor of mortality in patients at different stages of chronic kidney disease. Nephrol. Dial. Transpl. 25, 1183–1191. 10.1093/ndt/gfp592 19914995

[B78] Lim D. Song M. (2019). Development of bacteria as diagnostics and therapeutics by genetic engineering. J. Microbiol. 57 (8), 637–643. 10.1007/s12275-019-9105-8 31079333

[B79] Liu W. C. Tomino Y. Lu K. C. (2018). Impacts of indoxyl sulfate and p-cresol sulfate on chronic kidney disease and mitigating effects of AST-120. Toxins (Basel) 10 (9), 367. 10.3390/toxins10090367 30208594 PMC6162782

[B80] Liu Y. Li Y. Xu L. (2022). Quercetin attenuates podocyte apoptosis of diabetic nephropathy through targeting EGFR sig-naling. Front. Pharmacol. 12. 10.3389/fphar.2021.792777 PMC8766833 35069207

[B81] Liu Y. Peng X. Hu Z. Yu M. Fu J. Huang Y. (2021). Fabrication of a novel nitrogen-containing porous carbon adsorbent for pro-tein-bound uremic toxins removal. Mater Sci. Eng. C Mater Biol. Appl. 121, 111879. 10.1016/j.msec.2021.111879 33579500

[B82] Lu Z. Zeng Y. Lu F. Liu X. Zou C. (2015). Rhubarb enema attenuates renal tubulointerstitial fibrosis in 5/6 nephrectomized rats by alleviating indoxyl sulfate overload. PLoS One 10 (12), e0144726. 10.1371/journal.pone.0144726 26671452 PMC4684395

[B83] Luft F. C. (2008). Management of volume depletion and established acute renal failure. Therapy in nephrology and hypertension. Third Edition, 3–12.

[B84] Ma J. Li Y. Yang X. (2023). Signaling pathways in vascular function and hypertension: molecular mechanisms and therapeutic interventions. Signal Transduct. Target Ther. 8 (1), 168. 10.1038/s41392-023-01430-7 37080965 PMC10119183

[B85] Madero M. Cano K. B. Campos I. Tao X. Maheshwari V. Brown J. (2019). Removal of protein-bound uremic toxins during hemodialysis using a binding competitor. Clin. J. Am. Soc. Nephrol. 14 (3), 394–402. 10.2215/CJN.05240418 30755453 PMC6419294

[B86] Magnani S. Atti M. (2021). Uremic toxins and blood purification: a review of current evidence and future perspectives. Toxins 13 (4), 246. 10.3390/toxins13040246 33808345 PMC8066023

[B87] Mair R. D. Sirich T. L. Plummer N. S. Meyer T. W. (2018). Characteristics of colon-derived uremic solutes. Clin. J. Am. Soc. Nephrol. CJASN 13 (9), 1398–1404. 10.2215/CJN.03150318 30087103 PMC6140561

[B88] Malberti F. (2013). Hyperphosphataemia: treatment options. Drugs 73 (7), 673–688. 10.1007/s40265-013-0054-y 23625273

[B89] Mallipattu S. K. He J. C. Uribarri J. (2012). Role of advanced glycation endproducts and potential therapeutic interventions in dialysis patients. Semin. Dial. 25 (5), 529–538. 10.1111/j.1525-139X.2012.01081.x 22548330 PMC5558608

[B90] Marzocco S. Fazeli G. Di Micco L. Autore G. Adesso S. Dal Piaz F. (2018). Supplementation of short-chain fatty acid, sodium propionate, in patients on maintenance hemodialysis: beneficial effects on inflammatory parameters and gut-derived uremic toxins, A pilot study (plan study). J. Clin. Med. 7 (10), 315. 10.3390/jcm7100315 30274359 PMC6210519

[B91] McOrist A. L. Miller R. B. Bird A. R. Keogh J. B. Noakes M. Topping D. L. (2011). Fecal butyrate levels vary widely among individuals but are usually increased by a diet high in resistant starch. J. Nutr. 141 (5), 883–889. 10.3945/jn.110.128504 21430242

[B92] Meijers B. Toussaint N. D. Meyer T. Bammens B. Verbeke K. Vanrenterghem Y. (2011). Reduction in protein-bound solutes unacceptable as marker of dialysis efficacy during alternate-night nocturnal hemodialysis. Am. J. Nephrol. 34 (3), 226–232. 10.1159/000330176 21791919

[B93] Meyer T. W. Hostetter T. H. (2007). Urem. N. Engl. J. Med. 357 (13), 1316–1325. 10.1056/NEJMra071313 17898101

[B94] Mishima E. Fukuda S. Mukawa C. Yuri A. Kanemitsu Y. Matsumoto Y. (2017). Evaluation of the impact of gut microbiota on uremic solute accumulation by a CE-TOFMS-based metabolomics approach. Kidney Int. 92 (3), 634–645. 10.1016/j.kint.2017.02.011 28396122

[B95] Miyazaki T. Ise M. Seo H. Niwa T. (1997). Indoxyl sulfate increases the gene expressions of TGF-beta 1, TIMP-1 and pro-alpha 1(I) collagen in uremic rat kidneys. Kidney Int. Suppl. 62, S15–S22. 9350672

[B96] Mo Y. Sun H. Zhang L. (2021). Microbiome-Metabolomics analysis reveals the protection mechanism of α-ketoacid on ade-nine-induced chronic kidney disease in rats. Front. Pharmacol. 12, 657827. 10.3389/fphar.2021.657827 34045965 PMC8144710

[B97] Muirhead E. E. Reid A. F. (1948). A resin artificial kidney. J. Lab. Clin. Med. 33 (7), 841–844. 18869571

[B98] Nagata D. Yoshizawa H. (2020). Pharmacological actions of indoxyl sulfate and AST-120 that should Be recognized for the strategic treatment of patients with chronic kidney disease. Int. J. Nephrol. Renov. Dis. 13, 359–365. 10.2147/IJNRD.S287237 PMC7726832 33311993

[B99] Nakada Y. Onoue K. Nakano T. Ishihara S. Kumazawa T. Nakagawa H. (2019). AST-120, an oral carbon absorbent, protects against the progression of atherosclerosis in a mouse chronic renal failure model by preserving sFlt-1 expression levels. Sci. Rep. 9 (1), 15571. 10.1038/s41598-019-51292-9 31666542 PMC6821698

[B100] National Kidney Foundation (2003). K/DOQI clinical practice guidelines for bone metabolism and disease in chronic kidney disease. Am. J. Kidney Dis. 42 (4 Suppl. 3), S1–S201. 14520607

[B101] Neirynck N. Vanholder R. Schepers E. Eloot S. Pletinck A. Glorieux G. (2012). An update on uremic toxins. Int. Urol. Nephrol. 45, 139–150. 10.1007/s11255-012-0258-1 22893494

[B102] Nguyen Huu D. Dao Bui Quy Q. Nguyen Thi Thu H. (2021). A combination of hemodialysis with hemoperfusion helped to reduce the cardiovascular-related mortality rate after a 3-year follow-up: a pilot study in Vietnam. Blood Purif. 50 (1), 65–72. 10.1159/000507912 32615576

[B103] Niwa T. Emoto Y. Maeda K. Uehara Y. Yamada N. Shibata M. (1991). Oral sorbent suppresses accumulation of albumin-bound indoxyl sulphate in serum of haemodialysis patients. Nephrol. Dial. Transpl. 6 (2), 105–109. 10.1093/ndt/6.2.105 1906999

[B104] Niwa T. Ise M. (1994). Indoxyl sulfate, a circulating uremic toxin, stimulates the progression of glomerular sclerosis. J. Lab. Clin. Med. 124 (1), 96–104. 8035108

[B105] Ouellet G. Bouchard J. Ghannoum M. Decker B. S. (2014). Available extracorporeal treatments for poisoning: overview and limitations. Semin. Dial. 27 (4), 342–349. 10.1111/sdi.12238 24697909

[B106] Pandey K. R. Naik S. R. Vakil B. V. (2015). Probiotics, prebiotics and synbiotics-a review. J. Food Sci. Technol. 52 (12), 7577–7587. 10.1007/s13197-015-1921-1 26604335 PMC4648921

[B107] Pham N. M. Recht N. S. Hostetter T. H. Meyer T. W. (2008). Removal of the protein-bound solutes indican and p-cresol sulfate by peri-toneal dialysis. Clin. J. Am. Soc. Nephrol. 3 (1), 85–90. 10.2215/CJN.02570607 18045861 PMC2390983

[B108] Popkov V. A. Silachev D. N. Zalevsky A. O. Zorov D. B. Plotnikov E. Y. (2019). Mitochondria as a source and a target for uremic toxins. Int. J. Mol. Sci. 20 (12), 3094. 10.3390/ijms20123094 31242575 PMC6627204

[B109] Ramezani A. Massy Z. A. Meijers B. Evenepoel P. Vanholder R. Raj D. S. (2016). Role of the gut microbiome in uremia: a potential therapeutic target. Am. J. kidney Dis. official J. Natl. Kidney Found. 67 (3), 483–498. 10.1053/j.ajkd.2015.09.027 PMC5408507 26590448

[B110] Ramezani A. Raj D. S. (2014a). The gut microbiome, kidney disease, and targeted interventions. J. Am. Soc. Nephrol. JASN. 25 (4), 657–670. 10.1681/ASN.2013080905 24231662 PMC3968507

[B111] Ramezani A. Raj D. S. (2014b). The gut microbiome, kidney disease, and targeted interventions. J. Am. Soc. Nephrol. 25 (4), 657–670. 10.1681/ASN.2013080905 24231662 PMC3968507

[B112] Ren Q. Wang B. Guo F. (2022). Natural flavonoid pectolinarigenin alleviated hyperuricemic nephropathy via suppressing tgfβ/SMAD3 and JAK2/STAT3 signaling pathways. Front. Pharmacol. 12, 792139. 10.3389/fphar.2021.792139 35153751 PMC8829971

[B113] Rhee C. M. Ahmadi S. F. Kovesdy C. P. Kalantar-Zadeh K. (2018). Low-protein diet for conservative management of chronic kidney disease: a systematic review and meta-analysis of controlled trials. J. Cachexia Sarcopenia Muscle 9 (2), 235–245. 10.1002/jcsm.12264 29094800 PMC5879959

[B114] Roberts D. M. Buckley N. A. (2007). Pharmacokinetic considerations in clinical toxicology: clinical applications. Clin. Pharmacokinet. 46 (11), 897–939. 10.2165/00003088-200746110-00001 17922558

[B115] Rocchetti M. T. Cosola C. di Bari I. Magnani S. Galleggiante V. Scandiffio L. (2020). Efficacy of divinylbenzenic resin in removing indoxyl sulfate and P-cresol sulfate in hemodialysis patients: results from an *in vitro* study and an *in vivo* pilot trial (xuanro4-Nature 3.2). Toxins (Basel) 12 (3), 170. 10.3390/toxins12030170 32164382 PMC7150912

[B116] Rossi M. Johnson D. W. Xu H. Carrero J. J. Pascoe E. French C. (2015). Dietary protein-fiber ratio associates with circulating levels of indoxyl sulfate and p-cresyl sulfate in chronic kidney disease patients. Nutr. Metab. Cardiovasc Dis. 25 (9), 860–865. 10.1016/j.numecd.2015.03.015 26026209

[B117] Rossi M. Klein K. Johnson D. W. Campbell K. L. (2012). Pre-pro-and synbiotics: do they have a role in reducing uremic toxins? A systematic review and meta-analysis. Int. J. Nephrol. 2012, 673631. 10.1155/2012/673631 23316359 PMC3536316

[B118] Rowland I. Gibson G. Heinken A. Scott K. Swann J. Thiele I. (2018). Gut microbiota functions: metabolism of nutrients and other food components. Eur. J. Nutr. 57 (1), 1–24. 10.1007/s00394-017-1445-8 PMC5847071 28393285

[B119] Ryu J. H. Yu M. Lee S. Ryu D. R. Kim S. J. Kang D. H. (2016). AST-120 improves microvascular endothelial dysfunction in end-stage renal disease patients receiving hemodialysis. Yonsei Med. J. 57 (4), 942–949. 10.3349/ymj.2016.57.4.942 27189289 PMC4951472

[B120] Saito Y. Sato T. Nomoto K. Tsuji H. (2018). Identification of phenol- and p-cresol-producing intestinal bacteria by using media supplemented with tyrosine and its metabolites. FEMS Microbiol. Ecol. 94 (9), fiy125. 10.1093/femsec/fiy125 29982420 PMC6424909

[B121] Sato E. Saigusa D. Mishima E. Uchida T. Miura D. Morikawa-Ichinose T. (2017). Impact of the oral adsorbent AST-120 on organ-specific accumulation of uremic toxins: LC-MS/MS and MS imaging techniques. Toxins (Basel). 10 (1), 19. 10.3390/toxins10010019 29283413 PMC5793106

[B122] Schroeder B. O. Bäckhed F. (2016). Signals from the gut microbiota to distant organs in physiology and disease. Nat. Med. 22 (10), 1079–1089. 10.1038/nm.4185 27711063

[B123] Schulman G. Vanholder R. Niwa T. (2014). AST-120 for the management of progression of chronic kidney disease. Int. J. Nephrol. Renov. Dis. 7, 49–56. 10.2147/IJNRD.S41339 PMC3912158 24501542

[B124] Shen Y. Wang Y. Shi Y. Bi X. Xu J. Zhu Q. (2020). Improving the clearance of protein-bound uremic toxins using cationic liposomes as an ad-sorbent in dialysate. Colloids Surfaces B Biointerfaces. 186, 110725. 10.1016/j.colsurfb.2019.110725 31862563

[B125] Snelson M. Coughlan M. T. (2019). Dietary advanced glycation end products: digestion, metabolism and modulation of gut mi-crobial ecology. Nutrients 11 (2), 215. 10.3390/nu11020215 30678161 PMC6413015

[B126] Splendiani G. Albano V. Tancredi M. Daniele M. Pignatelli F. (1987). Our experience with combined hemodialysis-hemoperfusion treatment in chronic uremia. Biomater. Artif. Cells Artif. Organs 15 (1), 175–181. 10.3109/10731198709118517 3449135

[B127] Srisawat N. Tungsanga S. Lumlertgul N. Komaenthammasophon C. Peerapornratana S. Thamrongsat N. (2018). The effect of polymyxin B hemoperfusion on modulation of human leukocyte antigen DR in severe sepsis patients. Crit. Care 22 (1), 279. 10.1186/s13054-018-2077-y 30367647 PMC6204024

[B128] Stinghen A. E. Massy Z. A. Vlassara H. Striker G. E. Boullier A. (2016). Uremic toxicity of advanced glycation end products in CKD. J. Am. Soc. Nephrol. 27 (2), 354–370. 10.1681/ASN.2014101047 26311460 PMC4731113

[B129] Stremke E. R. Hill Gallant K. M. (2018). Intestinal phosphorus absorption in chronic kidney disease. Nutrients 10 (10), 1364. 10.3390/nu10101364 30249044 PMC6213936

[B130] Su P. Y. Lee Y. H. Kuo L. N. (2021). Efficacy of AST-120 for patients with chronic kidney disease: a network meta-analysis of randomized controlled trials. Front. Pharmacol. 12, 676345. 10.3389/fphar.2021.676345 34381357 PMC8350440

[B131] Susantitaphong P. Siribamrungwong M. Jaber B. L. (2013). Convective therapies versus low-flux hemodialysis for chronic kidney failure: a meta-analysis of randomized controlled trials. Nephrol. Dial. Transpl. 28 (11), 2859–2874. 10.1093/ndt/gft396 24081858

[B132] Takayama F. Taki K. Niwa T. (2003). Bifidobacterium in gastro-resistant seamless capsule reduces serum levels of indoxyl sulfate in patients on hemodialysis. Am. J. Kidney Dis. 41 (3 Suppl. 1), S142–S145. 10.1053/ajkd.2003.50104 12612972

[B133] Taki K. Takayama F. Niwa T. (2005). Beneficial effects of Bifidobacteria in a gastroresistant seamless capsule on hyperhomocyste-inemia in hemodialysis patients. J. Ren. Nutr. 15 (1), 77–80. 10.1053/j.jrn.2004.09.028 15648012

[B134] Tange Y. Takesawa S. Yoshitake S. (2015). Dialysate with high dissolved hydrogen facilitates dissociation of indoxyl sulfate from albumin. Nephrourol. Mon. 7 (2), e26847. 10.5812/numonthly.26847 25883914 PMC4393549

[B135] Toussaint N. D. Pedagogos E. Lioufas N. M. (2020). A randomized trial on the effect of phosphate reduction on vascular end points in CKD (IMPROVE-CKD). J. Am. Soc. Nephrol. 31 (11), 2653–2666. 10.1681/ASN.2020040411 32917784 PMC7608977

[B136] Vanholder R. De Smet R. Glorieux G. (2003). Review on uremic toxins: classification, concentration, and interindividual vari-ability. Kidney Int. 63 (5), 1934–1943. 10.1046/j.1523-1755.2003.00924.x 12675874

[B137] Vanholder R. Van Laecke S. Glorieux G. (2008). What is new in uremic toxicity? Pediatr. Nephrol. 23 (8), 1211–1221. 10.1007/s00467-008-0762-9 18324423 PMC2441592

[B138] Vaziri N. D. Wong J. Pahl M. Piceno Y. M. Yuan J. DeSantis T. Z. (2013). Chronic kidney disease alters intestinal microbial flora. Kidney Int. 83 (2), 308–315. 10.1038/ki.2012.345 22992469

[B139] Verkman A. S. (2006). Aquaporins in endothelia. Kidney Int. 69 (7), 1120–1123. 10.1038/sj.ki.5000226 16508658

[B140] Viaene L. Annaert P. de Loor H. Poesen R. Evenepoel P. Meijers B. (2013). Albumin is the main plasma binding protein for indoxyl sulfate and p-cresyl sulfate. Biopharm. Drug Dispos. 34 (3), 165–175. 10.1002/bdd.1834 23300093

[B141] Wang I. K. Wu Y. Y. Yang Y. F. Ting I. W. Lin C. C. Yen T. H. (2015). The effect of probiotics on serum levels of cytokine and endotoxin in peritoneal dialysis patients: a randomised, double-blind, placebo-controlled trial. Benef. Microbes 6 (4), 423–430. 10.3920/BM2014.0088 25609654

[B142] Wang J. Li R. Deng Z. (2018). Xueshuantong for injection ameliorates diabetic nephropathy in a rat model of streptozoto-cin-induced diabetes. Chin. J. Physiol. 61 (6), 349–359. 10.4077/CJP.2018.BAH637 30580505

[B143] Wang S. Fang Y. Yu X. Guo L. Zhang X. Xia D. (2019). The flavonoid-rich fraction from rhizomes of Smilax glabra Roxb. ameliorates renal oxidative stress and inflammation in uric acid nephropathy rats through promoting uric acid excretion. Biomed. Pharmacother. 111, 162–168. 10.1016/j.biopha.2018.12.050 30579255

[B144] Wang X. Yang S. Li S. (2020). Aberrant gut microbiota alters host metabolome and impacts renal failure in humans and rodents. Gut 69 (12), 2131–2142. 10.1136/gutjnl-2019-319766 32241904 PMC7677483

[B145] Wang Y. Dong Q. Hu S. Decoding microbial genomes to understand their functional roles in human complex diseases. iMeta.(n/a):1, e14. 10.1002/imt2.14 PMC10989872 38868571

[B146] Wei J. Li R. Zhang P. (2023). Efficient selective removal of uremic toxin precursor by olefin-linked covalent organic frameworks for nephropathy treatment. Nat. Commun. 14 (1), 2805. 10.1038/s41467-023-38427-3 37193688 PMC10188479

[B147] Wu I. W. Hsu K. H. Lee C. C. Sun C. Y. Hsu H. J. Tsai C. J. (2011). p-Cresyl sulphate and indoxyl sulphate predict progression of chronic kidney disease. Nephrol. Dial. Transpl. 26 (3), 938–947. 10.1093/ndt/gfq580 PMC3042976 20884620

[B148] Wyss M. Kaddurah-Daouk R. (2000). Creatine and creatinine metabolism. Physiol. Rev. 80 (3), 1107–1213. 10.1152/physrev.2000.80.3.1107 10893433

[B149] Yamamoto S. Sato M. Sato Y. Wakamatsu T. Takahashi Y. Iguchi A. (2018). Adsorption of protein-bound uremic toxins through direct hemoperfusion with hexadecyl-immobilized cellulose beads in patients undergoing hemodialysis. Artif. Organs 42 (1), 88–93. 10.1111/aor.12961 28703401

[B150] Yang J. Lim S. Y. Ko Y. S. (2019). Intestinal barrier disruption and dysregulated mucosal immunity contribute to kidney fibrosis in chronic kidney disease. Nephrol. Dial. Transpl. 34 (3), 419–428. 10.1093/ndt/gfy172 29939312

[B151] Yoshida H. Yokoyama K. Munakata K. Maruyama Y. Yamamoto R. Hanaoka K. (2007). Superior dialytic clearance of beta2 microglobulin and p-cresol by high-flux hemodialysis as compared to peritoneal dialysis. Kidney Int. 71 (5), 467–468. author reply 467-468. 10.1038/sj.ki.5002063 17315011

[B152] Yu H-X. Lin W. Yang K. (2021). Transcriptome-based network analysis reveals hirudin potentiates anti-renal fibrosis ef-ficacy in UUO rats. Front. Pharmacol. 12, 741801. 10.3389/fphar.2021.741801 34621173 PMC8490886

[B153] Yu J. Deng M. Zhao J. Huang L. (2010). Decreased expression of klotho gene in uremic atherosclerosis in apolipoprotein E-deficient mice. Biochem. Biophys. Res. Commun. 391 (1), 261–266. 10.1016/j.bbrc.2009.11.046 19912987

[B154] Zazzeroni L. Pasquinelli G. Nanni E. Cremonini V. Rubbi I. (2017). Comparison of quality of life in patients undergoing hemodi-alysis and peritoneal dialysis: a systematic review and meta-analysis. Kidney Blood Press Res. 42 (4), 717–727. 10.1159/000484115 29049991

[B155] Zelante T. Iannitti R. G. Cunha C. De Luca A. Giovannini G. Pieraccini G. (2013). Tryptophan catabolites from microbiota engage aryl hydrocarbon receptor and balance mucosal reactivity via interleukin-22. Immunity 39 (2), 372–385. 10.1016/j.immuni.2013.08.003 23973224

[B156] Zheng W. Huang T. Tang Q-Z. Li S. Qin J. Chen F. (2021). Astragalus polysaccharide reduces blood pressure, renal damage, and dysfunction through the TGF-β1-ILK pathway. Front. Pharmacol. 12. 10.3389/fphar.2021.706617 PMC8527034 34690754

[B157] Zou Y. T. Zhou J. Zhu J. H. (2022). Gut microbiota mediates the protective effects of traditional Chinese medicine formula qiong-yu-gao against cisplatin-induced acute kidney injury. Microbiol. Spectr. 10, e0075922. 10.1128/spectrum.00759-22 35481834 PMC9241845

